# Rapid and sensitive detection of *bla*_KPC_ gene in clinical isolates of *Klebsiella pneumoniae* by a molecular real-time assay

**DOI:** 10.1186/2193-1801-2-31

**Published:** 2013-01-30

**Authors:** Adriana Mosca, Luisa Miragliotta, Raffaele Del Prete, Gerasimos Tzakis, Lidia Dalfino, Francesco Bruno, Laura Pagani, Roberta Migliavacca, Aurora Piazza, Giuseppe Miragliotta

**Affiliations:** 1 Department of Interdisciplinary Medicine Microbiology, University of Bari, Piazza Giulio Cesare, Bari, 70124 Italy; 2 Department of Emergency and Organ Transplantation Anesthesia and Intensive Care Unit, University of Bari, Piazza Giulio Cesare, Bari, 70124 Italy; 3 Microbiology, University of Pavia, via Brambilla 74, Pavia, 27100 Italy

**Keywords:** *Klebsiella pneumoniae*, Carbapenem resistance, *bla*_KPC_, NASBA™

## Abstract

**Background:**

The aim of this study was the rapid identification of *bla*_KPC_ gene in 38 *Klebsiella pneumoniae* clinical isolates with reduced susceptibility to carbapenems. The modified Hodge Test (MHT) was carried out to phenotypically determine whether resistance to carbapenems was mediated by a carbapenemase. The detection of the *bla*_KPC_ gene was performed by real-time acid nucleic sequence-based amplification (NASBA™™), specifically designed for the detection of KPC RNA target.

**Results:**

Thirty-two/38 isolates evaluated by MHT showed the production of carbapenemases, while all the strains exhibited the production of KPC by inhibition test with phenylboronic acid (the combined disk test with IPM/IPM plus phenylboronic acid). The detection of *bla*_KPC_ gene by Nuclisens EasyQ KPC yielded positive results in 38/38 (100%) strains. The presence of *bla*_KPC_ gene was confirmed in all *K. pneumoniae* isolates when tested by the gold standard PCR assay.

**Conclusions:**

In consideration of the serious challenge represented by infections due to *K. pneumoniae* it appears necessary the rapid identification of carbapenemases in clinical settings as it is made possible by the use of NASBA™ assay.

## Introduction

Over the last decade carbapenemase-producing *Enterobacteriaceae* have emerged and these multidrug-resistant pathogens became a problem in the clinical care of patients. Among *Enterobacteriaceae, Klebsiella pneumoniae* carbapenemase (KPC)-producing strains of *K. pneumoniae* broadly disseminated worldwide (Nordmann et al. 2009). KPC is a beta-lactamase enzyme, classified as ESBL_CARBA-A,_ (Giske et al. 2009) encoded by *bla*_KPC_ gene, that confers resistance to all beta-lactam antibiotics including carbapenems. Misidentification of KPC-producing bacteria is common with standard susceptibility testing (Nordmann et al. 2009), whereas the presence of a KPC may cause MIC elevations that remain within the susceptible or intermediate range. Therefore, although time-consuming, phenotypic confirmation tests (i.e. modified Hodge test (MHT) and carbapenemase inhibitor test) have been recommended (Clinical and Laboratory Standards Institute 2009). Inappropriate treatment may be the consequence of inaccurate detection of KPCs, resulting in compromised patients’ outcomes (Weisenberg et al. 2009). The emergence of metallo beta-lactamases (MBLs) producing *K. pneumoniae* strains further suggested the need to investigate the mechanism of resistance for a more rapid infection control perspective (Vatopoulos 2008). In order to control the spread of *bla*_KPC_ –containing bacteria in hospitalized patients, an important role may be played by a rapid and sensitive *bla*_KPC_ diagnostic tools which help in isolating colonized or infected patients. In this report, we evaluated the performance of a new molecular assay (NASBA™, NucliSens EasyQKPC, bioMérieux, France), for the rapid detection of *bla*_KPC_ gene in isolates of *K. pneumoniae* from patients hospitalized in ICU, as well as in Medical and Surgical wards of the teaching hospital Policlinico of Bari, Bari, Italy.

## Materials and methods

*Clinical isolates.* A total of 38 non duplicate clinical isolates of *K. pneumoniae* resistant to carbapenems [i.e. imipenem (IPM), meropenem (MEM), ertapenem (ERT)] were included in this study. The isolates were collected in a period of four months (September to December 2011) from separate patients who were hospitalized in the teaching hospital Policlinico of Bari, Bari, Italy. Microorganisms were isolated from multiple infection sites, including blood (n = 10), urine (n = 11), bronchial aspirate (n = 10), rectal swabs (n = 3), throat swab (n = 1), sputum (n = 2), and bile (n = 1) specimens. *K. pneumoniae* (n = 4), *Pseudomonas aeruginosa* (n = 2), *Acinetobacter* spp. (n = 2), and *Escherichia coli* (n = 2) with no known resistance were included as negative control.

*Antimicrobial susceptibility determination and carbapenemases assays.* Detailed antimicrobial susceptibility was carried out automatically and interpreted according to the recommendations of European Committee for Antimicrobial Susceptibility testing (EUCAST) (Vading et al. 2011). All *K. pneumoniae* strains were identified as possible KPC producers by MicroScan Walkaway System using 43 MS GNC panels (Siemens, New York, NY) on the basis of resistance to IPM, MEM and ERT. The resistance level of *K. pneumoniae* isolates to IPM was confirmed by Etest (bioMérieux, France) according to the manufacturers’ instructions. All isolates were screened for the production of carbapenemases, using MHT which is a phenotypic test used to determine if resistance to carbapenems is mediated by a carbapenemase enzyme (Carvalhaes et al. 2010). This test was performed using both MEM and IPM 10 μg disks. The presence of a distorted inhibition zone after overnight incubation was interpreted as a positive test result. The isolates were further investigated by combined disk test with IPM and IPM plus phenylboronic acid (PBA) or ethylenediaminetetraacetic acid (EDTA) as inhibitors of KPC or MBLs, respectively (Tsakris et al. 2009). The stock solution of PBA was prepared by dissolving PBA (benzeneboronic acid; Sigma-Aldrich, Steinheim, Germany) in dimethylsulfoxide and water at a concentration of 20 mg/mL (Coudron 2005). From this solution 20 μL was dispensed onto IPM disks. The combined disk IPM/EDTA was purchased from Biolife, Italy. Inhibition tests were performed for the detection of ESBLs and therefore stock solution of PBA was also dispensed onto disks containing ceftazidime (CAZ) or cefotaxime (CTX) with and without clavulanate (CA) at the same final amount (i.e. 400 μg). All the antibiotic disks were commercially available. The tests were performed by inoculating Mueller Hinton agar plates with the standard disk diffusion method; an increase in the growth-inhibitory zone around the disk containing the added beta-lactamase inhibitor was observed. The test was considered positive for KPC or MBLs when the growth inhibitory zone around either the IPM/PBA or the IPM/EDTA disk was 5 mm or greater of the growth inhibitory zone diameter around the disk containing IPM alone. With regard to the detection of ESBLs, when the zone diameter of either CTX-CA or CAZ-CA disk tested in combination with PBA (CTX-CA-PBA or CAZ-CA-PBA, respectively) was 5 mm or greater of the zone diameter of CTX or CAZ containing PBA (CTX-PBA or CAZ-PBA, respectively) the test was considered positive. The presence of AmpC beta-lactamase was phenotypically tested by determining IPM MICs in agar with and without 200 μg/ml cloxacillin and by using the AmpC detection Etest strips (bioMérieux, France).

*Detection of bla*KPC *gene by molecular methods.* All *K. pneumoniae* isolates were also investigated by nucleic acid sequence-based amplification, NASBA™, NucliSens EasyQ KPC (bioMérieux, France), for the detection of the *bla*_KPC_ gene. This molecular method couples nucleic acid sequence-based amplification (NASBA) with real-time procedure assay. NASBA is a sensitive, isothermal, transcription-based amplification system designed specifically for the detection of KPC RNA target in real time mode. Nucleic acid amplification uses primers that are specific for KPC RNA sequences and for the synthetic KPC internal control RNA. Any KPC RNA present in the sample is co-amplified along with the internal control otherwise other nucleic acid sequences will not be amplified. According to the manufacturer’s instructions, a 0.5 McFarland suspension of each isolate was prepared from an overnight non selective culture plate and heated at 95°C for 5 min. The reaction mixture was prepared in the tube strip by transferring 2.5 μl of internal control solution, 2.5 μl of heated bacterial suspension, 10 μl of primers solution (including both primers, molecular beacon probes and nucleotides) and incubated for 2 minutes at 65°C and 2 minutes at 41°C. After addition of 5 μL of enzyme mix (AMV-RT, RNase H and T7 RNA polymerase, bovine serum albumin), amplification was followed for 90 minutes at 41°C in NucliSENSE EasyQ Analyzer (bioMérieux, France) according to the assay protocol. Results were validated for each isolate according to the amplification of the internal control.

In order to compare the results obtained by Nuclisens EasyQ KPC method with a gold standard molecular test all the 38/38 isolates, along with the negative control strains, were further investigated by PCR assay using the fol-lowing primers: *bla*KPC For: TGTCACTGTATCGCCGTC; e *bla*KPC Rev: CTCAGTGCTCTACAGAAAACC (Yigit et al. 2001). The amplification protocol consisted of a denaturation step at 95°C (5 min) followed by 35 cycles at 95°C for 60 sec, 55°C for 40 sec, 72°C for 90 sec; was also included a cycle of extension at 72°C for 10 min.

## Results

**Figure 1:**
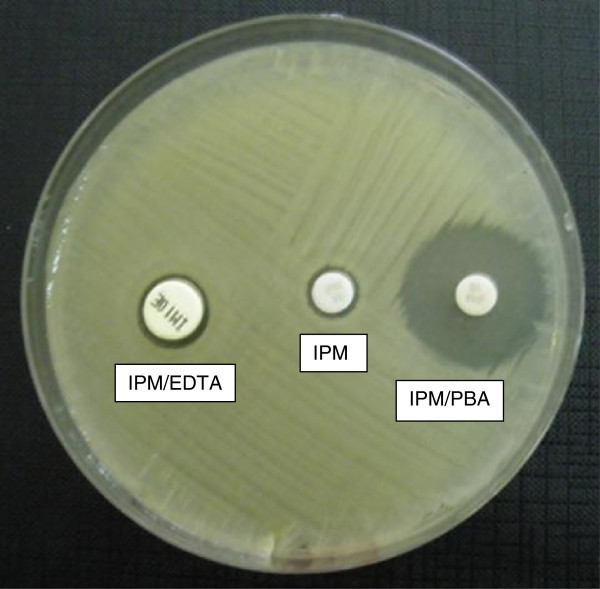
**Representative results of inhibition test with IPM, IPM/PBA, and IPM/EDTA for*Klebsiella pneumoniae*isolates.**

**Figure 2:**
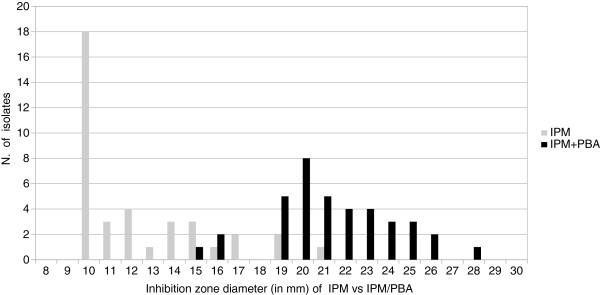
**Increase in the inhibition zone diameters of IPM/PBA disks versus those of IPM for 38*Klebsiella pneumoniae*isolates.**

All the *K. pneumoniae* strains exhibited the same pattern of antibiotic susceptibility (Table 1). The isolates showed resistance to all the antibiotics tested with the exception of gentamycin (MIC, < 2 μg/ml), tigecycline (MIC, < 1 μg/ml), fosfomycin (MIC, < 32 μg/ml), and tetracycline (MIC, ≤ 4 μg/ml). Carbapenems susceptibility carried out by automated testing revealed MICs >8 μg/ml for IPM and MEM, and >4 μg/ml for ERT, respectively. When IPM susceptibility was determined by Etest MIC_50_ and MIC_90_ values were >32 μg/ml (range 4 μg/ml-32 μg/ml). Thirty-two/38 (84%) strains evaluated by MHT showed the production of carbapenemase, regardless of carbapenems tested. Six/38 (16%) strains negative to MHT had IPM MIC values decreased (range 4 μg/ml-12 μg/ml). All the isolates were positive for the combined disk test with PBA, thus suggesting the production of KPC-type enzyme (Figure 1). Figure 2 shows the increase of the inhibition zone in the presence of IPM in comparison with the inhibition zone in the presence of IPM/PBA. In 4/38 strains the co-production of both KPC and MBLs was suggested by the difference of at least 7 mm between the diameters of inhibition zone of IPM and IPM/EDTA. Since the Ampc detection was negative in all the cases, the presence of plasmid-mediated Ampc was excluded. The CLSI confirmatory test in the presence of clavulanate/PBA was positive for CAZ and CTX, clearly indicating the co-production of ESBLs. The detection of *bla*_KPC_ gene by Nuclisens EasyQKPC yielded positive results in 38/38 (100%) cases. All control isolates resulted negative for either phenotypic or genotyping tests (Table 2).

**Table 1:** **Susceptibility profile of all*K. pneumoniae*isolates tested by automated system against different antimicrobial agents**

Antimicrobial agents	MIC (μg/ml)
Amikacin	> 32 R
Amoxicillin/ Clavulanate	> 16/8 R
Aztreonam	> 16 R
Cefepime	> 16 R
Cefotaxime	> 32 R
Ceftazidime	> 16 R
Cefuroxime	> 16 R
Ciprofloxacin	> 2 R
Ertapenem	> 4 R
Fosfomycin	≤ 32 S
Gentamicin	≤ 4 S
Imipenem	> 8 R
Levofloxacin	> 4 R
Meropenem	> 8 R
Piperacillin/Tazobactam	> 64 R
Tetracycline	≤ 4 S
Tigecycline	≤ 1 S
Tobramycin	> 8 R
Trimetoprim/Sulfametoxazole	> 2/38 R

S, susceptible; R, Resistant.

**Table 2:** **Phenotypic and genotypic detection of*Klebsiella pneumoniae*carbapenemase in clinical isolates**

Clinical isolates (No)	Modified Hodge Test (MHT)	Disk test		Nuclisens EasyQ KPC®	PCR assay
		BA	EDTA		
*K. pneumoniae (*38)	32/38 positive	38/38 positive	4/38 positive	38/38 positive	38/38 positive
	6/38 negative				
*K. pneumoniae* (4), *P. aeruginosa* (2), *Acinetobacter ssp.* (2), *E. coli* (2) with no known resistance	10/10 negative	10/10 negative	10/10 negative	10/10 negative	10/10 negative

In order to confirm the presence of *bla*KPC gene by a standard and well known molecular method, all the isolates were further investigated by PCR assay. According to the EasyQ KPC assay, all the strains resulted positive by PCR, thus confirming the presence of the *bla*KPC resistance determinant. Control isolates previously resulted negative (i.e. by phenotypic or genotyping tests) were also confirmed by PCR assay (Table 2).

## Discussion

Carbapenems are beta-lactam antibiotics with broad-spectrum antibacterial activity, often used to treat infections due to ESBL-producing Gram-negative bacteria (Paterson and Bonomo 2005). Resistance to carbapenems represents a serious problem in the treatment of such infections. In particular, KPCs enzymes are capable of hydrolyzing all known beta-lactam antibiotics and displaying resistance to beta-lactamase inhibitors. While the presence of ESBLs can be masked by the expression of KPC, the co-production of ESBLs may contribute to either the hydrolytic activity of KPC or the resistance to broad-spectrum cephalosporins (Nordmann et al. 2009). KPC genes are carried on a variety of plasmids and may be co-transferred with ESBL genes (Petrella et al. 2008; Tsakris et al. 2008). Moreover, both genes are often associated with plasmid mediated fluoroquinolone and aminoglycoside resistance determinants (Nordmann et al. 2009; Poirel et al. 2006). Therefore, the horizontal transmission of *bla*_KPC_ genes highly contributes to the dissemination of strains resistant to several classes of antibiotics leaving a few therapeutic choices. Over recent years, the spread of KPC-producing bacteria created the necessity to implement the laboratory with tests able to promptly report any carbapenem-resistant isolate to either the clinician for the appropriated antimicrobial therapy or the hospital infection control team for the appropriate contact isolation precautions. However, detection of carbapenem-resistant organisms may be problematic because some isolates express low levels of resistance that may not be detected by conventional methods (Landman et al. 2005; Anderson et al. 2007) and results still vary among different methods (Bulik et al. 2010). When carbapenems susceptibility was investigated by automated systems, we were precluded from comparing the accuracy of MICs above 8 μg/ml as it was done by using Etest for IPM susceptibility determination. On the other hand, susceptibility tests of carbapenemase producing bacteria using Etest are often difficult to interpret (Nordmann et al. 2009). Indeed, carbapenem-resistant bacteria incorrectly identified as carbapenem-susceptible have been reported, with the result of inappropriate selection of therapy (Anderson et al. 2007; Bratu et al. 2005; Tenover et al. 2006). MHT was also evaluated for detection of KPC-mediated resistance. This phenotypic test is sensitive for the detection of carbapenemases production but does not provide information regarding the type of enzyme involved. False-positive results have been indeed reported for CTX-M beta-lactamases producing strains with reduced outer membrane permeability (Carvalhaes et al. 2010; Pasteran et al. 2009). Some investigators have raised the problem of difficulties in the interpretation of the cloverleaf test for weak carbapenemase producers (Pasteran et al. 2009). In our study 6/38 (16%) strains negative to MHT had IPM MIC values decreased (range 4 μg/ml-12 μg/ml). The inhibition test with PBA allowed to detect the production of KPC, whereas EDTA positivity suggested the co-production of MBLs. Although in our hands the results were clear, it has been noted that the interpretation of inhibition test may be difficult and subjective in some cases (Drieux et al. 2008). In the light of the above considerations, it appears necessary to detect carbapenemases important from a clinical point of view by methods not impractical for studies involving large sample sizes, as well as for rapid identification in clinical settings. Molecular methods such as PCR and real time-PCR, for the identification of *bla*_KPC_ gene have been used principally in research laboratories and reference centers (Hindiyeh et al. 2008).

## Conclusions

We have herein described the performance of a nowadays commercially available test based on real time-NASBA, for the automated amplification detection of *bla*_KPC_ gene. Our results are consistent with those recently published which confirm the advantages of this test (Spanu et al. 2012). Aside from its sensitivity, it has indeed confirmed to facilitate results with significantly less time (4 hours) and labor, allowing an even more rapid detection of drug-resistant bacteria in clinical samples.
